# Diagnostic Performance of Mobile Low‐Field MRI and CT for Acute Headache as the Isolated Clinical Manifestation in the Neurology Emergency Department

**DOI:** 10.1002/cns.71082

**Published:** 2026-08-11

**Authors:** Xinyan Hu, Chong Han, Qianmei Jiang, Yue Suo, Wanliang Du, Zhe Zhang, Ning Wang, Bingshan Xue, Yihuai Wang, Zixiao Li, Xingquan Zhao, Hao Li, Tao Liu, Yongjun Wang, Jing Jing, Xuewei Xie

**Affiliations:** ^1^ Department of Neurology, Beijing Tiantan Hospital Capital Medical University Beijing China; ^2^ China National Clinical Research Center for Neurological Diseases Beijing China; ^3^ Tiantan Neuroimaging Center of Excellence Beijing China; ^4^ Emergency Department, Beijing Tiantan Hospital Capital Medical University Beijing China; ^5^ Department of Neurosurgery, Xuanwu Hospital Capital Medical University Beijing China; ^6^ Ray Plus Medical Technology Beijing China; ^7^ Beijing Advanced Innovation Center for Biomedical Engineering, School of Biological Science and Medical Engineering Beihang University Beijing China

**Keywords:** emergency department, headache, mobile low‐field magnetic resonance imaging, stroke

## Abstract

**Background:**

Headache may manifest as the sole or predominant presenting symptom of various types of acute stroke. While mobile low‐field magnetic resonance imaging (mLF‐MRI) shows promise for rapid and accurate diagnosis in the emergency department (ED), its diagnostic concordance using computed tomography (CT) as the reference standard requires clarification.

**Methods:**

This prospective study included 199 patients who presented to the ED of Beijing Tiantan Hospital with headache within 72 h of onset, without Face‐Arm‐Speech Test (FAST) symptoms, and who underwent 0.23‐T mLF‐MRI between January and July 2024. This study evaluated the diagnostic utility of 0.23‐T mLF‐MRI in emergency patients presenting with an isolated acute headache. We specifically compared the consistency of mLF‐MRI and CT in detecting subarachnoid hemorrhage (SAH) and other causes.

**Results:**

Of the 199 patients with headache who did not have FAST symptoms, 71 (35.68%) were diagnosed with stroke, including 60 cases of SAH and 2 patients with intracerebral hemorrhage (ICH). Notably, 9 patients presenting with headaches but without FAST symptoms were diagnosed with acute ischemic stroke. The diagnostic concordance between the fluid‐attenuated inversion recovery (FLAIR) sequence of mLF‐MRI and CT for SAH was excellent (κ = 0.99). The mLF‐MRI provided short scanning times and offered a portable, low‐power diagnostic tool.

**Conclusion:**

For emergency patients with headaches as the initial and isolated clinical manifestation, mLF‐MRI may serve as a supplementary diagnostic imaging modality that provides additional diagnostic information in selected ED settings. mLF‐MRI, especially FLAIR sequences, demonstrated high CT‐referenced diagnostic concordance for SAH and may also detect early ischemic lesions.

## Graphical abstract

For emergency patients with headaches as the initial and isolated clinical manifestation, mLF‐MRI may serve as a supplementary diagnostic imaging modality that provides additional diagnostic information in selected ED settings. mLF‐MRI, especially FLAIR sequences, demonstrates high CT‐referenced diagnostic concordance for SAH and can also detect early ischemic lesions.

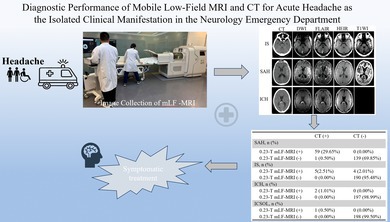

**Abbreviations**
CT: computed tomography
ED: emergency department
FLAIR: fluid‐attenuated inversion recovery
HEIR: hematoma‐enhanced inversion recovery
ICH: intracerebral hemorrhage
IS: ischemic stroke
mLF‐MRI: mobile low‐field magnetic resonance imaging
MRI: magnetic resonance imaging
SAH: subarachnoid hemorrhage
T1WI: T1‐weighted imaging

## 1 Introduction

Headache ranks as the fifth most common reason for emergency department (ED) visits [1], accounting for approximately 2% of all ED presentations [2]. This common symptom requires careful evaluation to distinguish between benign causes and conditions requiring urgent intervention. Headache may manifest as the sole or predominant presenting symptom in various types of acute stroke, including acute ischemic stroke (AIS), subarachnoid hemorrhage (SAH), and intracerebral hemorrhage (ICH) [3, 4]. Although migraine, particularly migraine with aura, has been epidemiologically associated with an increased risk of IS, this association is likely influenced by shared vascular risk factors, and classical primary migraine alone does not routinely warrant neuroimaging in the absence of clinical red flags [5], whereas acute cerebrovascular events may occasionally present with isolated headache, thereby necessitating timely imaging when secondary causes are suspected [6]. Disorders characterized by prominent headache symptoms may increase the risk of both ischemic and hemorrhagic stroke [7], underscoring the critical importance of timely imaging in guiding appropriate treatment decisions.

Computed tomography (CT) has become the most widely used initial diagnostic modality in the ED because of its short scanning time and wide availability [8]. However, early infarct signs on CT can be subtle and challenging to identify [9], potentially limiting its sensitivity in the initial hours following symptom onset [10]. Furthermore, when a headache persists for more than 6 h or when a small amount of sulcal bleeding is present, the sensitivity of CT decreases [11]. Conversely, high‐field magnetic resonance imaging (MRI) offers superior differentiation among AIS, ICH, and SAH compared with CT [12]. Despite its diagnostic advantages, conventional high‐field MRI systems operating at 1.5–3 Tesla (T) necessitate prolonged acquisition times and impose stringent patient selection criteria, including strict safety protocols, stable clinical conditions, and the need for specialized operators. These requirements limit MRI availability in ED and resource‐constrained settings [13]. To address these limitations, mobile low‐field magnetic resonance imaging (mLF‐MRI) systems operating at 0.23‐T mLF‐MRI have recently been developed. 0.23‐T mLF‐MRI offers several potential advantages, including shorter scanning times; lower overall power, cooling, and magnetic field shielding requirements; and the ability to be operated in hospital wards or regular ED settings [14, 15, 16].

Prior experimental and clinical studies have demonstrated that 0.23‐T mLF‐MRI can rapidly and accurately differentiate between stroke types, including cerebral hemorrhage and infarction, supporting its clinical use with shorter scan times [13]. Furthermore, previous diagnostic case–control studies have indicated that high‐field fluid‐attenuated inversion recovery (FLAIR) sequences offer sensitivity comparable to or superior to CT for detecting acute or subacute SAH [17, 18]. Despite these promising findings, the diagnostic efficacy of 0.23‐T mLF‐MRI‐FLAIR sequences compared with CT in prospective clinical studies specifically involving patients with headache remains largely unexamined, highlighting a critical gap in current research regarding mLF‐MRI's application in emergency settings.

Therefore, we aimed to prospectively compare mLF‐MRI with CT for detecting various causes of acute headache, particularly when headache is the initial and isolated clinical manifestation. Our primary hypothesis was that mLF‐MRI would provide complementary diagnostic information to CT in patients presenting with isolated acute headache. We further hypothesized that mLF‐MRI would demonstrate high diagnostic concordance with CT for SAH detection while providing additional capability for detecting acute ischemic lesions.

## 2 Methods

### 2.1 Study Design and Participants

**FIGURE 1:**
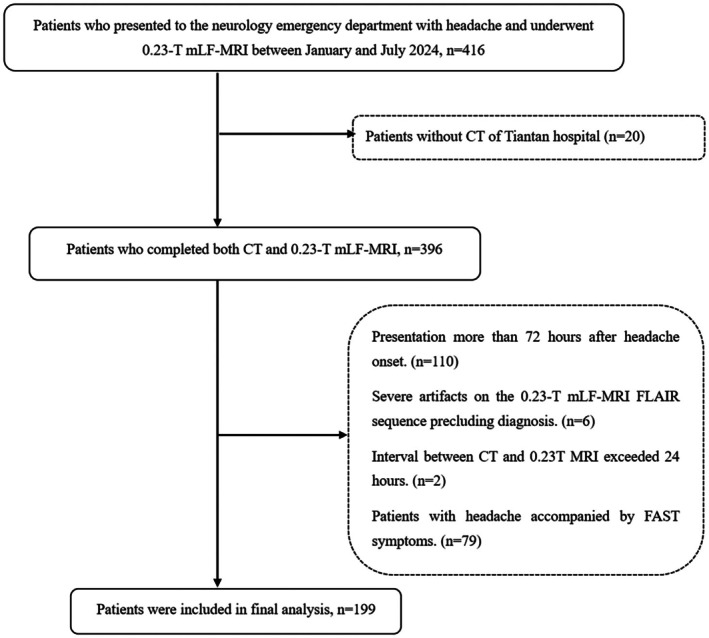
Flowchart of the study. CT, computed tomography; FLAIR, fluid‐attenuated inversion recovery; mLF‐MRI, mobile low‐field magnetic resonance imaging.

We prospectively screened consecutive patients who presented to the neurology ED of Beijing Tiantan Hospital, a teaching hospital in Beijing, China, with acute headache and underwent 0.23‐T mLF‐MRI between January 2024 and July 2024. The 0.23‐T mLF‐MRI examination was performed as part of this prospective study after written informed consent was obtained from the patient or a legally authorized representative and the absence of MRI contraindications was confirmed. A total of 416 patients were initially screened. During the initial screening stage, patients were excluded if they did not undergo CT at Beijing Tiantan Hospital (*n* = 20), leaving 396 patients who completed both CT and 0.23‐T mLF‐MRI. Additional exclusions were applied to patients who presented more than 72 h after headache onset (*n* = 110), those with severe artifacts on the 0.23‐T mLF‐MRI FLAIR sequence that precluded diagnosis (*n* = 6), those in whom the interval between CT and mLF‐MRI exceeded 24 h (*n* = 2), and patients with headache accompanied by FAST symptoms (face‐arm‐speech test, *n* = 79) [19]. Finally, a total of 199 patients were included in the final analysis. A flowchart of the study is shown in Figure 1.

### 2.2 Neuroimaging Protocol

The study utilized mLF‐MRI (ACUTA Elfin; Ray Plus Medical Technology Co. Ltd., Foshan, China) installed in the ED of Beijing Tiantan Hospital. As reported in previous studies [13, 20, 21], the mLF‐MRI scanner used in this study is characterized by compact physical dimensions, measuring 160 cm in height and 120 cm in width, with a weight of 2400 kg. This system operates efficiently on a standard 220‐V power supply, which represents a significant advantage because it avoids the substantial power and electrical current requirements of MRI scanners with higher magnetic field strengths that necessitate supercooling cryogens. The system design incorporates a patient gurney capable of supporting up to 150 kg and an optimized 25.8 × 20 cm head coil to enhance imaging capability. The technical specifications of the system include a 25 mT/m gradient amplitude and a 60 mT/m/ms slew rate, which are specifically engineered to enable expedited neuroimaging in critical emergency settings.

The acquisition protocol included the following five sequences: Fluid‐attenuated inversion recovery T1‐weighted (FLAIR T1) (repetition time (TR)/echo time (TE) = 1326.0/24.4 ms, acquisition time = 1 min and 32 s), FLAIR T2 (TR/TE = 4380.0/107.9 ms, acquisition time = 1 min and 32 s), T2WI (TR/TE = 2500.0/150.4 ms, acquisition time = 1 min and 33 s), and DWI (TR/TE = 2598.2/100.3 ms, acquisition time = 4 min and 22 s). Meanwhile, the mLF‐MRI incorporates a novel hematoma‐enhanced inversion recovery (HEIR, TR/TE = 1100.7/24.4 ms, acquisition time = 1 min and 17 s) sequence. The detailed imaging parameters are provided in Table S1. For comparative analysis, non‐contrast CT scans were performed in the orbitomeatal plane, with 5‐mm‐thick slices encompassing the entire neurocranium from the skull base to the vertex.

### 2.3 Image Interpretation and Diagnostic Criteria

SAH was defined as hyperintensity in the subarachnoid spaces, including cerebral sulci and basal cisterns on FLAIR sequences [22]. Diagnostic criteria for IS on 0.23‐T mLF‐MRI were hyperintensity on DWI and isointensity on the HEIR sequence [13]. Figure S1 shows bedside mLF‐MRI examination in the emergency department with representative imaging examples of IS, SAH, and ICH. CT was used to classify stroke types according to established reference standards.

Images obtained using 0.23‐T mLF‐MRI and CT were analyzed by two expert neuroradiologists and two expert stroke neurologists who were not involved in patient care and were blinded to all clinical information and to each other's assessments. Prior to image interpretation, all readers completed a calibration session using representative cases from an independent dataset to ensure consistent application of the diagnostic criteria. The readers independently reviewed the images and recorded the presence of IS, ICH, SAH, and isolated headaches. Digital images were presented using commercially available software that allowed adjustment of contrast, brightness, and image size. All the images were anonymized and contained no patient identifiers. During image evaluation, readers were allowed to adjust image brightness and contrast as needed. CT and 0.23‐T mLF‐MRI images were interpreted independently and in separate reading sessions, with readers blinded to the results of the other imaging modality as well as to all clinical information. To minimize recall bias, CT and mLF‐MRI examinations from the same patient were reviewed on different days and presented in a fully randomized order, without any pairing or cross‐referencing between modalities. For each imaging modality, a case was considered positive for a given diagnosis only when at least three of the four independent readers reached concordant interpretations. In cases of a 2:2 or 3:1 split, a final diagnosis was determined by consensus through group discussion. Inter‐reader agreement before consensus adjudication for CT and 0.23‐T mLF‐MRI sequences is summarized in Table S3.

### 2.4 Statistical Analysis

Continuous variables were expressed as medians with interquartile ranges (IQRs). Categorical variables are presented as counts and percentages and were compared using the chi‐square test. For diagnostic performance analysis, 2 × 2 contingency tables were constructed to compare the diagnostic outcomes of mLF‐MRI and CT. The sensitivity and specificity of mLF‐MRI for detecting SAH were calculated using CT as the reference standard. Concordance between mLF‐MRI and CT was assessed using McNemar's test for paired proportions. Inter‐reader agreement among the four independent readers was assessed separately for each imaging modality (CT, FLAIR, and DWI sequences) using Fleiss' kappa statistics for categorical variables (presence or absence of each imaging finding) prior to consensus adjudication. The 95% confidence intervals for kappa were calculated using asymptotic standard errors. Kappa values were interpreted according to standard guidelines, with values < 0 indicating poor agreement, 0–0.20 slight, 0.21–0.40 fair, 0.41–0.60 moderate, 0.61–0.80 substantial, and > 0.80 excellent agreement. Subgroup analyses were performed based on the time from symptom onset to the mLF‐MRI examination. All statistical analyses were performed using IBM SPSS Statistics for Windows (version 27.0; IBM Corporation, Armonk, NY, USA) and R Studio (version 4.2.2; RStudio Inc., Boston, MA, USA). A two‐sided *p* value < 0.05 was considered statistically significant.

## 3 Results

### 3.1 Participant Characteristics

Baseline patient characteristics are summarized in Table 1. In the final analysis of 199 patients (median age, 58 years; IQR = 44–67), 71 patients (35.68%) were diagnosed with stroke. This group includes 60 patients (30.15%) with SAH, 2 patients (1.01%) with ICH, and 9 patients (4.52%) with AIS. In addition, 1 patient (0.50%) was diagnosed with an intracranial space‐occupying lesion (ICSOL). Regarding the timing of imaging, 112 patients (56.28%) underwent CT within 24 h of symptom onset, whereas the remaining 87 patients (43.72%) underwent CT between 24 and 72 h after onset. Detailed baseline characteristics of all patients are presented in Table 1.

**TABLE 1:** Baseline characteristics of patients with headache as the isolated clinical manifestation.

Characteristics	All patients, *n* = 199
Age, years	58 (44, 67)
Male, *n* (%)	92 (46.23)
Medical history	
Hypertension, *n* (%)	84 (42.21)
Diabetes, *n* (%)	34 (17.09)
Hyperlipidemia, *n* (%)	19 (9.55)
Coronary heart disease, *n* (%)	15 (7.54)
Onset time to CT, hours	
Total, hours	14.88 (5.28, 47.10)
< 24 h, *n* (%)	112 (56.28)
24–72 h, *n* (%)	87 (43.72)
Onset Time to 0.23‐T mLF‐MRI, hours	
Total, hours	24.55 (9.18, 48.15)
< 24 h, *n* (%)	93 (46.73)
24–72 h, *n* (%)	106 (53.26)
Interval between CT and 0.23‐T MRI, hours	1.00 (0.32, 6.92)

*Note:* Categorical variables are presented as number (%) and continuous variables are presented as median (interquartile range).

Abbreviations: CT, computed tomography; ICSOL, intracranial space‐occupying lesion; MRI, magnetic resonance imaging; SAH, subarachnoid hemorrhage.

The 0.23‐T mLF‐MRI demonstrated high concordance with CT for the diagnosis of SAH, IS, ICH, and ICSOL. Among patients with SAH detected on CT, only one case of mild hemorrhage was missed by mLF‐MRI. For IS, mLF‐MRI detected 9 cases, whereas CT detected only 5 cases, according to the diagnostic criteria. Additionally, one case of an intracranial space‐occupying lesion was identified in a patient presenting with headache. Table 2 presents the paired proportions of diagnoses obtained using CT and mLF‐MRI in patients with isolated headaches.

**TABLE 2:** Paired proportion analysis of CT and mobile low‐field MRI in patients with isolated headache.

	CT (+)	CT (−)
SAH, *n* (%)		
0.23‐T mLF‐MRI (+)	59 (29.65%)	0 (0.00%)
0.23‐T mLF‐MRI (−)	1 (0.50%)	139 (69.85%)
IS, *n* (%)		
0.23‐T mLF‐MRI (+)	5 (2.51%)	4 (2.01%)
0.23‐T mLF‐MRI (−)	0 (0.00%)	190 (95.48%)
ICH, *n* (%)		
0.23‐T mLF‐MRI (+)	2 (1.01%)	0 (0.00%)
0.23‐T mLF‐MRI (−)	0 (0.00%)	197 (98.99%)
ICSOL, *n* (%)		
0.23‐T mLF‐MRI (+)	1 (0.50%)	0 (0.00%)
0.23‐T mLF‐MRI (−)	0 (0.00%)	198 (99.50%)

Abbreviations: CT, computed tomography; ICSOL, intracranial space‐occupying lesion; IS, ischemic stroke; mLF‐MRI, mobile low‐field magnetic resonance imaging; SAH, subarachnoid hemorrhage.

### 3.2 mLF‐MRI Versus CT Panel Results

**FIGURE 2:**
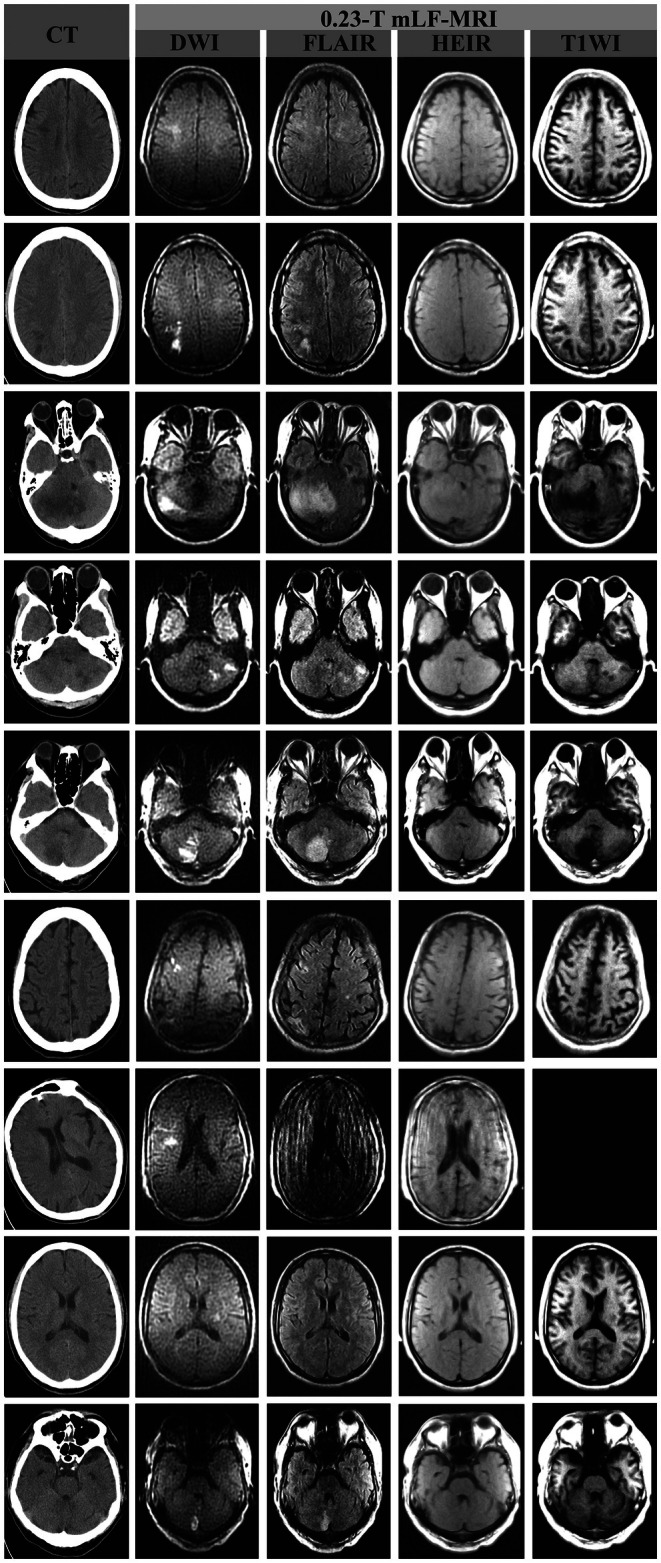
IS diagnosed in 9 patients presenting only with headache. CT, computed tomography; DWI, diffusion‐weighted imaging; FLAIR, fluid‐attenuated inversion recovery; HEIR, hematoma enhanced inversion recovery; IS, ischemic stroke; mLF‐MRI, mobile low‐field magnetic resonance imaging; T1WI, T1‐weighted imaging.

**FIGURE 3:**
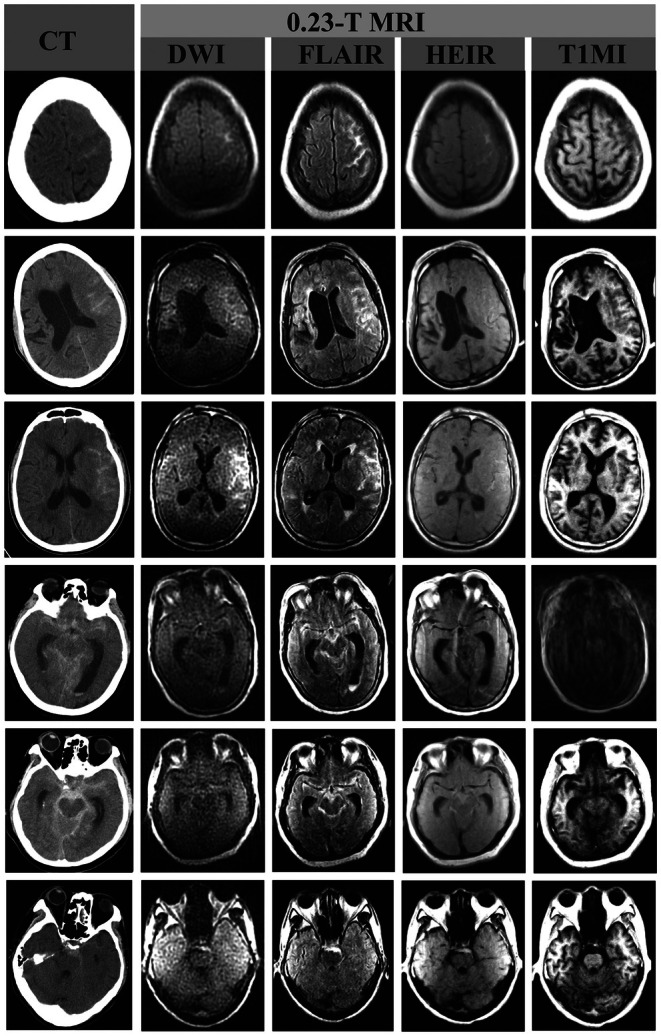
Representative cases of subarachnoid hemorrhage. CT, computed tomography; DWI, diffusion‐weighted imaging; FLAIR, fluid‐attenuated inversion recovery; HEIR, hematoma enhanced inversion recovery; mLF‐MRI, mobile low‐field magnetic resonance imaging; T1WI, T1‐weighted imaging.

**FIGURE 4:**
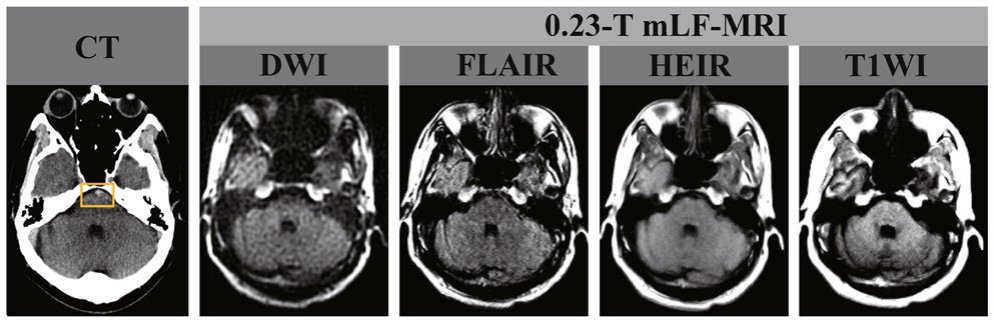
Special case of subarachnoid hemorrhage detected by CT but not visualized on 0.23 T mLF‐MRI. Case presentation: The patient presented with acute headache without focal neurological deficits. CT performed approximately 50 h after symptom onset demonstrated subarachnoid hemorrhage, and mLF‐MRI was subsequently completed approximately 19 h after the CT examination. CT, computed tomography; DWI, diffusion‐weighted imaging; FLAIR, fluid‐attenuated inversion recovery; HEIR, hematoma enhanced inversion recovery; mLF‐MRI, mobile low‐field magnetic resonance imaging; T1WI, T1‐weighted imaging.

Figure 2 illustrates the mLF‐MRI and CT images of 9 patients with AIS who presented with headaches. Representative cases of SAH are shown in Figure 3. Additionally, Figure 4 illustrates a unique case of SAH identified by CT but not by 0.23‐T mLF‐MRI in the study and its corresponding case characteristics. Other mLF‐MRI and CT images of patients with SAH are presented in Figure S2.

The comparison of mLF‐MRI and CT findings is presented in Table 3. The FLAIR sequence demonstrated excellent diagnostic agreement for SAH, with a κ value of 0.99 (95% CI: 0.97–1.00). In comparison, the diagnostic agreement of the DWI sequence for SAH was lower, with a κ value of 0.66 (95% CI: 0.54–0.78). Stratified analysis based on the time interval from symptom onset to 0.23‐T mLF‐MRI revealed consistent diagnostic performance across time categories (< 24 h from onset: κ = 1.00; 24–72 h from onset: κ = 0.98).

**TABLE 3:** Paired proportions of CT and mobile low‐field MRI in SAH diagnosis.

SAH	CT (+)	CT (−)	Kappa (95% CI)	*p*
Total				
0.23‐T FLAIR (+)	59 (98.33)	0 (0.00)	0.99 (0.97, 1.00)	> 0.99
0.23‐T FLAIR (−)	1 (1.67)	139 (100.00)		
0.23‐T DWI (+)	35 (58.33)	0 (0.00)	0.66 (0.54, 0.78)	< 0.001
0.23‐T DWI (−)	25 (41.67)	139 (100.00)		
< 24 h from onset time to 0.23‐T mLF‐MRI^a^				
0.23‐T FLAIR (+)	26 (100.00)	0 (0.00)	1.00 (1.00, 1.00)	> 0.99
0.23‐T FLAIR (−)	0 (0.00)	67 (100.00)		
0.23‐T DWI (+)	16 (61.54)	0 (0.00)	0.70 (0.52, 0.88)	0.002
0.23‐T DWI (−)	10 (38.46)	67 (100.00)		
24–72 h from onset time to 0.23‐T mLF‐MRI^a^				
0.23‐T FLAIR (+)	33 (97.06)	0 (0.00)	0.98 (0.94, 1.00)	> 0.99
0.23‐T FLAIR (−)	1 (2.94)	72 (100.00)		
0.23‐T DWI (+)	19 (55.88)	0 (0.00)	0.63 (0.47, 0.79)	< 0.001
0.23‐T DWI (−)	15 (44.11)	72 (100.00)		

Abbreviations: CT, computed tomography; DWI, diffusion weighted imaging; FLAIR, fluid attenuated inversion recovery; mLF‐MRI, mobile low‐field magnetic resonance imaging.

^a^ Symptoms to scan means from stroke symptoms onset to low field 0.23‐T MRI scan.

Supplement Table 2 summarizes the diagnostic performance of FLAIR and DWI sequences for SAH detection, using CT as the reference. FLAIR sensitivity was 100.0% in patients scanned less than 24 h after symptom onset and 98.33% in those scanned between 24 and 72 h, whereas DWI sensitivity was lower across both intervals. The specificities of FLAIR and DWI were 100.00% in all subgroups.

## 4 Discussion

Neuroimaging plays a crucial role in evaluating patients presenting with headache, particularly those without FAST symptoms. In this prospective study, a subset of patients presenting with isolated headaches was found to harbor AIS. The mLF‐MRI FLAIR sequence demonstrated strong diagnostic concordance with CT for detecting SAH, whereas DWI showed lower concordance when compared separately with CT. The FLAIR sequence showed excellent agreement with CT and maintained stable diagnostic performance across different time intervals from symptom onset. Furthermore, mLF‐MRI effectively detected AIS and ICH using DWI and HEIR sequences. These findings, derived from ED presentations of headache, may be directly applicable to clinical practice.

Headache is a common presenting symptom in patients with acute cerebrovascular events and may occasionally represent the only initial manifestation of AIS [23, 24]. Previous studies have highlighted that headaches of a new type or with altered characteristics are causally related to ischemic stroke and should not be underestimated in the ED setting [23]. Our research revealed that patients presenting with isolated headache without FAST symptoms may still suffer from AIS, which is consistent with previously published studies [25]. Despite this, current clinical practice prioritizes non‐contrast head CT as the first‐line imaging modality for suspected stroke due to its rapid acquisition and widespread availability. However, conventional CT has limited sensitivity in the early phase of ischemic stroke, particularly within the first few hours of symptom onset, and misdiagnosis or underdiagnosis remains a significant clinical concern [26, 27]. Headache complaints account for a substantial proportion of national ED visits [28, 29], since most headache complaints evaluated in emergency settings are benign.

Previous studies have shown that mLF‐MRI has the potential to differentiate cerebral hemorrhage from cerebral infarction with both speed and accuracy [13]. This study further demonstrates that 0.23‐T mLF‐MRI shows reliable diagnostic performance for SAH. Earlier 0.5 T MRI case–control studies from the early 1990s also demonstrated the diagnostic reliability of FLAIR sequences for SAH, findings that are consistent with the present results [18, 22]. FLAIR detects SAH by nullifying the signal from normal cerebrospinal fluid (CSF) while preserving the signal from hemorrhagic CSF because of its shortened T1 relaxation time. Additionally, because acute subarachnoid blood retains a relatively long T2 relaxation time compared with brain parenchyma, it appears hyperintense on FLAIR images. This allows clear visualization of the SAH, even when blood is diluted within the CSF. Collectively, our results further validate previous evidence that FLAIR imaging is a reliable modality for detecting SAH.

Previous studies have demonstrated that high‐field MRI (1.5‐T) and CT are highly sensitive and specific modalities for the diagnosis of SAH [27]. However, MRI acquisition times are relatively long, which limits the routine application of MRI in emergency settings, where rapid and reliable diagnosis is of utmost importance [30]. Meanwhile, a major limitation of conventional CT within the first few hours of symptom onset is its limited sensitivity for identifying early cerebral ischemia [12]. Low‐field MRI offers several practical advantages that may substantially improve clinical workflows, particularly in resource‐limited settings. These systems are more accessible and are often more convenient for critically ill patients who cannot be easily transported [31]. Moreover, low‐field MRI retains the capability to detect acute ischemic infarction, thereby providing comprehensive neuroimaging in the acute setting, where differentiation between hemorrhagic and ischemic stroke is essential for ensuring appropriate management of patients presenting with headache [13, 20].

Our findings suggest that mLF‐MRI, particularly FLAIR sequences, demonstrates diagnostic sensitivity for SAH comparable to that of CT, while also providing the advantage of early ischemia detection. Moreover, previous studies have suggested that the diagnostic performance and clinical utility of MRI in patients with mild or isolated symptoms remain clinically important [32] and previous studies also have shown that mLF‐MRI enables rapid differentiation between cerebral hemorrhage and cerebral infarction and may provide additional diagnostic information beyond that obtained from CT alone in selected patients [13, 33]. These characteristics indicate that low‐field MRI may serve as a valuable adjunctive imaging modality in the ED, particularly for patients presenting with acute headaches of unclear etiology, in whom the possibility of an underlying stroke should be carefully considered. Integration of low‐field MRI into acute diagnostic pathways may improve diagnostic assessment in selected patients presenting with isolated headache [31].

This study introduced a novel mLF‐MRI system capable of early SAH diagnosis with high accuracy and rapid scanning time using integrated sequences, including FLAIR and DWI. This technology has the potential to enhance diagnostic accuracy and refine headache management by facilitating rapid differentiation between cerebral hemorrhage and cerebral infarction. The mLF‐MRI system operates on standard power, presenting an effective solution for diagnostic imaging in the ED. Several limitations of this study should be acknowledged. First, this single‐center study in a tertiary hospital may have limited generalizability to other patients, such as those presenting with isolated headache, dizziness, or vertigo in general emergency settings. Small AIS/ICH numbers and temporal imaging changes may affect subgroup analyses, warranting validation in larger multicenter studies. Second, the study population was limited to emergency department patients presenting with headache within 72 h of symptom onset, and detailed headache phenotyping was not systematically recorded, limiting applicability to prolonged or other clinical presentations. Third, although excellent concordance between mLF‐MRI and CT was observed for SAH detection, detailed assessment of small‐volume or anatomically subtle hemorrhages was limited by the small number of discordant cases. Finally, cost‐effectiveness and economic evaluation of mLF‐MRI compared with existing diagnostic strategies were not assessed and warrant further investigation.

## 5 Conclusion

For emergency patients presenting with headache as the initial and isolated clinical manifestation, mLF‐MRI may serve as a supplementary diagnostic tool in the emergency department. In particular, mLF‐MRI, especially FLAIR sequences, provides diagnostic performance for SAH comparable to that of CT and may also detect early ischemic lesions, thereby providing complementary information for the etiological evaluation of headache. This imaging approach may serve as a valuable adjunct in selected ED settings.

## Author Contributions

X.X. and J.J. had full access to all of the data in the study and take responsibility for the integrity of the data and the accuracy of the data analysis. Concept and design: X.X. and J.J. Acquisition, analysis, or interpretation of data: X.X., J.J., and Y.W. Drafting of the manuscript: X.H., C.H., and X.X. Critical review of the manuscript for important intellectual content: X.X. and J.J. Statistical analysis: X.H., C.H., and Q.J. Obtained funding: X.X., J.J. Administrative, technical, or material support: Y.S., W.D., Z.Z., N.W., B.X., Y.W., Z.L., X.Z., H.L., T.L. Supervision: Z.L., X.Z., H.L., T.L., Y.W., J.J., X.X.

## Funding

Supported by the Noncommunicable Chronic Diseases‐National Science and Technology Major Project of China (Grants 2024ZD0527600 and 2024ZD0527604), National Natural Science Foundation of China (82471356), and National Natural Science Foundation of China (Grant 82271329).

## Ethics Statement

This study was approved by the Ethics Committee and the Institutional Review Board (IRB) of Beijing Tiantan Hospital, Capital Medical University (IRB approval number: KY‐2023‐286‐03). This study was conducted in accordance with the tenets of the Declaration of Helsinki. Written informed consent was obtained from all participants before enrollment.

## Conflicts of Interest

Authors B.X. and Y.W. are employees of Ray Plus Medical Technology, Beijing, China, which develops mobile low‐field MRI systems. The other authors declare no conflicts of interest. No additional financial or non‐financial conflicts of interest exist among the authors.

## Supporting information


**Figure S1:** Bedside examination using mobile low‐field magnetic resonance imaging in the emergency department.

**Figure S2:** Other mLF‐MRI and CT images of patients with SAH. CT, computed tomography; DWI, diffusion‐weighted imaging; FLAIR, fluid‐attenuated inversion recovery; HEIR, hematoma enhanced inversion recovery; mLF‐MRI, mobile low‐field magnetic resonance imaging; SAH, subarachnoid hemorrhage; T1WI, T1‐weighted imaging.

**Table S1:** Mobile low‐field MRI sequence details.
**Table S2:** Sensitivity and specificity of blinded imaging diagnosis by time from onset to low field 0.23‐T MRI scan among patients with SAH.
**Table S3:** Inter‐reader agreement among four readers for CT and 0.23 T mLF‐MRI sequences.

## Data Availability

The raw data supporting the conclusions of this article will be made available by the corresponding author.
