# Supplementary material for: Diagnostic Performance of Mobile Low‐Field MRI and CT for Acute Headache as the Isolated Clinical Manifestation in the Neurology Emergency Department
Source: CNS Neurosci Ther. 2026 Aug 11;32(8):e71082. doi: 10.1002/cns.71082 (PMC13459070; doi:10.1002/cns.71082)
Supplement: Supplementary file 1 [file CNS-32-e71082-s001.pdf]

Supplement figure 2. Other mLF-MRI and CT images of patients with SAH.

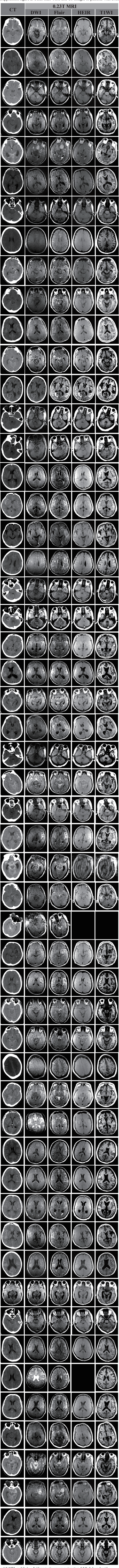

mLF-MRI, mobile low-field magnetic resonance imaging; SAH, Subarachnoid hemorrhage; DWI, Diffusion-Weighted Imaging; FLAIR, Fluid-Attenuated Inversion Recovery; HEIR, Hematoma Enhanced Inversion Recovery; T1WI, T1-Weighted Imaging; CT, Computed Tomography
