# Supplementary material for: Diagnostic Performance of Mobile Low‐Field MRI and CT for Acute Headache as the Isolated Clinical Manifestation in the Neurology Emergency Department
Source: CNS Neurosci Ther. 2026 Aug 11;32(8):e71082. doi: 10.1002/cns.71082 (PMC13459070; doi:10.1002/cns.71082)
Supplement: Supplementary file 2 [file CNS-32-e71082-s003.pdf]

Supplement figure 1. Bedside examination using mobile low-fieldmagnetic resonance imaging in the emergency department.

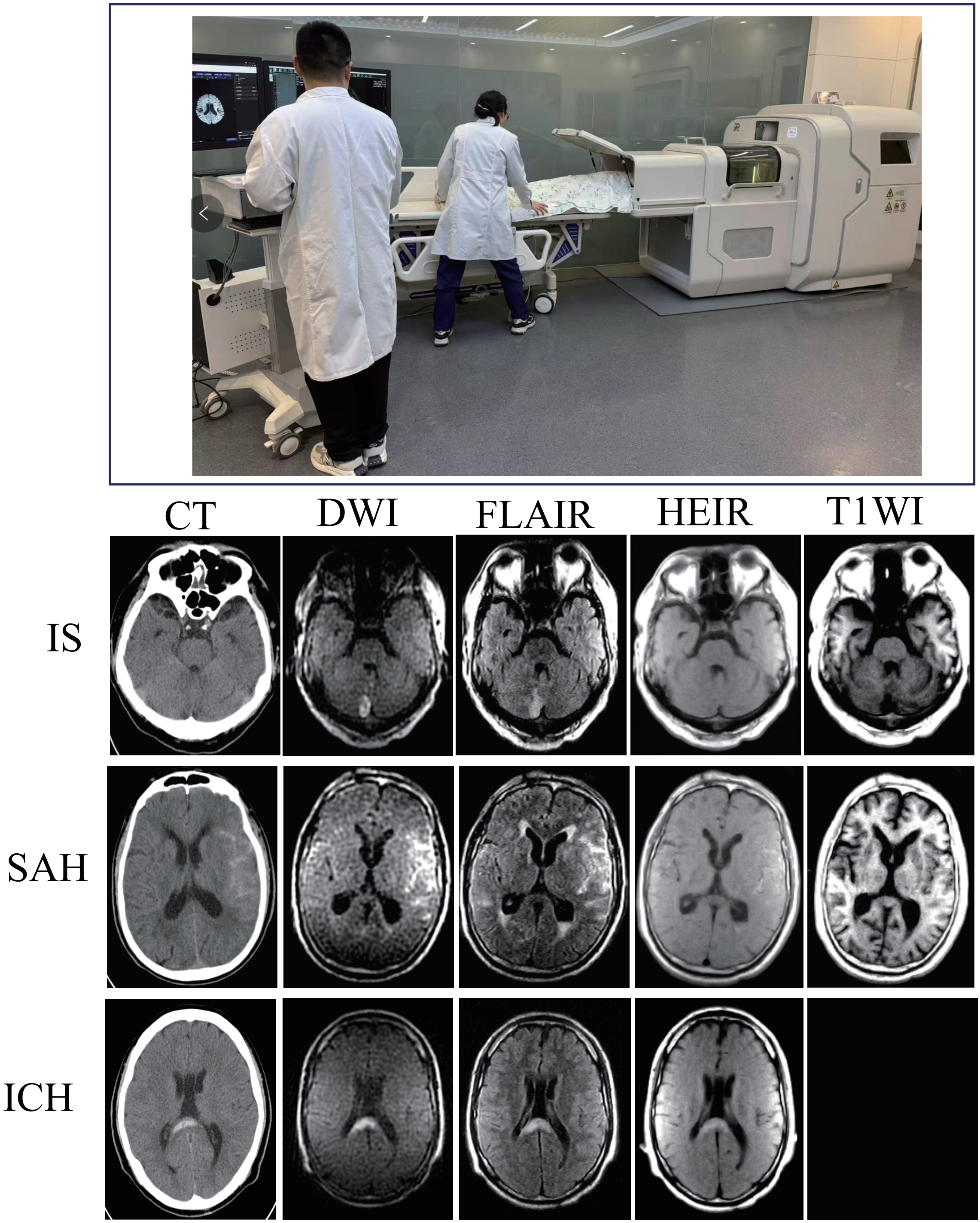

IS,cases of acute ischemic stroke; SAH, cases of subarachnoid hemorrhage;
ICH,cases of intracranial hemorrhage, TIWI, T1-weighted imaging;
FLAIR, fluid-attenuated inversion recovery; HEIR, hematoma enhanced inversion recovery;
DWI,diffusion-weighted imaging; CT, Computed Tomography
