# Supplementary material for: Diagnostic Performance of Mobile Low‐Field MRI and CT for Acute Headache as the Isolated Clinical Manifestation in the Neurology Emergency Department
Source: CNS Neurosci Ther. 2026 Aug 11;32(8):e71082. doi: 10.1002/cns.71082 (PMC13459070; doi:10.1002/cns.71082)
Supplement: Supplementary file 3 [file CNS-32-e71082-s002.docx]

**Diagnostic Performance of Mobile Low-Field MRI and CT for Acute Headache as the Isolated Clinical Manifestation in the Neurology Emergency Department**

Xinyan Hu, MD*; Chong Han, MD*; Qianmei Jiang, MD; Yue Suo, MD; Wanliang Du, MD; Zhe Zhang, PhD; Ning Wang, MD; Bingshan Xue, PhD; Yihuai Wang, PhD; Zixiao Li, MD; Xingquan Zhao, MD; Hao Li, MD, PhD; Tao Liu, PhD; Yongjun Wang, MD; Jing Jing, MD, PhD^#^; Xuewei Xie MD, PhD^#^

Supplement table 1, Mobile low-field MRI sequence details.

Supplement table 2, Sensitivity and specificity of blinded imaging diagnosis by time from onset to low field 0.23-T MRI scan among patients with SAH.

Supplement table 3, Inter-reader agreement among four readers for CT and 0.23T mLF-MRI sequences.

Supplement figure 1. Bedside examination using mobile low-fieldmagnetic resonance imaging in the emergencydepartment.

Supplement figure 2. Other mLF-MRI and CT images of patients with SAH.

Supplement table 1 Mobile low-field MRI sequence details.

| Sequence | Resolution, slice thickness/slice gap/total slices (mm) | Sequence Parameters | Matrix | Average Acquisition Time (min) | b value (s/mm2) |
| --- | --- | --- | --- | --- | --- |
| Localizer | 6/2/5 | TR/TE = 60.0/5.7 msec | 96×64 | 12″ |  |
| Axi FLAIRT1 | 7/2/13 | TR/TE/TI = 1326.0/25.5/425.0 msec | 256×192 | 1′32″ |  |
| Axi FLAIRT2 | 7/2/13 | TR/TE/TI = 4380.0/107.9/1600 msec | 256×160 | 1′32″ |  |
| Axi FSET2 | 7/2/13 | TR/TE = 2500.0/105.4 msec | 256×192 | 1′33″ |  |
| HEIR | 7/2/13 | TR/TE = 1100.7/24.4 msec | 256×192 | 1′17″ |  |
| DWI | 7/2/13 | TR/TE = 2598.2/100.3 msec | 128×128 | 4′22″ | 1000 |

TR, repetition time; TE, echo time; TI, inversion time; FLAIR, fluid-attenuated inversion recovery; HEIR, hematoma enhanced inversion recovery; DWI, diffusion-weighted imaging

Supplement table 2. Sensitivity and specificity of blinded imaging diagnosis by time from onset to low field 0.23-T MRI scan among patients with SAH.

| SAH | 0.23-T Flair | 0.23-T DWI |
| --- | --- | --- |
| Sensitivity |  |  |
| Total | 98.33% (91.06, 99.96) | 58.33% (44.88,70.93) |
| <24 hours | 100.00% (86.77, 100.00) | 61.54% (40.57,79.77) |
| 24-72 hours | 97.06% (84.67.81,99.93) | 55.88% (37.89,72.81) |
| Specificity |  |  |
| Total | 100.00% (97.38,100.00) | 100.00% (97.38, 100.00) |
| <24 hours | 100.00% (94.64,100.00) | 100.00% (94.64, 100.00) |
| 24-72 hours | 100.00% (95.01, 100.00) | 100.00% (95.01, 100.00) |
| Accuracy |  |  |
| Total | 99.50% (97.23, 99.99) | 90.29% (82.02, 91.70) |
| <24 hours | 100.00% (96.11, 100.00) | 89.25% (81.11, 94.72) |
| 24-72 hours | 99.06% (94.86, 99.98) | 85.85% (77.74, 91.86) |

CT, computed tomography; DWI, Diffusion weighted imaging; Flair, Fluid attenuated inversion recovery;

*Symptoms to scan means from stroke symptoms onset to low field 0.23-T MRI scan.

Supplement table 3. Inter-reader agreement among four readers for CT and 0.23T mLF-MRI sequences

|  | Kappa | 95%CT |
| --- | --- | --- |
| CT | 1.00 | (0.95, 1.00) |
| 0.23T-FLAIR | 0.98 | (0.93, 1.00) |
| 0.23T-DWI | 0.92 | (0.87,0.96) |

mLF-MRI, mobile low-field magnetic resonance imaging; CT, computed tomography; DWI, Diffusion weighted imaging; Flair, Fluid attenuated inversion recovery
